# Supplementary material for: Innovation in an E. coli evolution experiment is contingent on maintaining adaptive potential until competition subsides
Source: PLoS Genet. 2018 Apr 12;14(4):e1007348. doi: 10.1371/journal.pgen.1007348 (PMC5918244; doi:10.1371/journal.pgen.1007348)
Supplement: S1 Table — Clade designations describe placement in the phylogenetic tree of all sequenced strains from the population and relative to key evolutionary transitions in this population: UC, Unsuccessful Clade; C1, Clade 1; C2, Clade 2; C3, Clade 3; C3+, Clade 3 Cit+; C3+H, Clade 3 Cit+ hypermutator. (DOCX) [file pgen.1007348.s006.docx]

| **Strain** | **Generation** | **Clade** | **Reference** |
| --- | --- | --- | --- |
| REL764A | 500 |  | Tenaillon et al. 2016 |
| REL764B | 500 |  | Tenaillon et al. 2016 |
| REL966A | 1,000 |  | Tenaillon et al. 2016 |
| REL966B | 1,000 |  | Tenaillon et al. 2016 |
| REL1070A | 1,500 |  | Tenaillon et al. 2016 |
| REL1070B | 1,500 |  | Tenaillon et al. 2016 |
| REL1166A | 2,000 |  | Blount et al. 2012 |
| REL1166B | 2,000 |  | Tenaillon et al. 2016 |
| REL2181A | 5,000 |  | Tenaillon et al. 2016 |
| ZDB409 | 5,000 |  | Blount et al. 2012 |
| REL4538A | 10,000 | UC | Tenaillon et al. 2016 |
| ZDB1 | 10,000 |  | This study |
| ZDB425 | 10,000 |  | This study |
| ZDB429 | 10,000 | UC | Blount et al. 2012 |
| REL7179B | 15,000 |  | Tenaillon et al. 2016 |
| ZDB445 | 15,000 |  | This study |
| ZDB446 | 15,000 | UC | Blount et al. 2012 |
| ZDB458 | 20,000 |  | Blount et al. 2012 |
| ZDB464 | 20,000 |  | Blount et al. 2012 |
| ZDB467 | 20,000 |  | Blount et al. 2012 |
| ZDB477 | 25,000 | C1 | Blount et al. 2012 |
| ZDB478 | 25,000 | C3 | This study |
| ZDB483 | 25,000 | C3 | Blount et al. 2012 |
| ZDB486 | 25,000 | C3 | This study |
| ZDB488 | 25,000 | C3 | This study |
| ZDB309 | 27,000 | C3 | This study |
| ZDB310 | 27,000 | C3 | This study |
| ZDB317 | 27,000 | C3 | This study |
| ZDB334 | 28,000 | C3 | This study |
| ZDB339 | 28,000 | C3 | This study |
| ZDB13 | 29,000 | C3 | This study |
| ZDB14 | 29,000 | C3 | This study |
| ZDB16 | 30,000 | C1 | Blount et al. 2012 |
| ZDB17 | 30,000 | C3 | This study |
| ZDB18 | 30,000 | C3 | This study |
| ZDB357 | 30,000 | C2 | Blount et al. 2012 |
| ZDB19 | 30,500 | C3 | This study |
| ZDB20 | 30,500 | C3 | This study |
| ZDB23 | 31,000 | C3 | This study |
| ZDB25 | 31,500 | C3 | This study |
| ZDB27 | 31,500 | C3 | This study |
| ZDB199 | 31,500 | C1 | Blount et al. 2012 |
| ZDB200 | 31,500 | C2 | Blount et al. 2012 |
| ZDB564 | 31,500 | C3+ | Blount et al. 2012 |
| ZDB30 | 32,000 | C3+ | Blount et al. 2012 |
| ZDB172 | 32,000 | C3+ | Blount et al. 2012 |
| ZDB143 | 32,500 | C2 | Blount et al. 2012 |
| ZDB158 | 32,500 | C2 | Blount et al. 2012 |
| CZB152 | 33,000 | C3+ | Blount et al. 2012 |
| CZB154 | 33,000 | C3+ | Blount et al. 2012 |
| CZB199 | 33,000 | C1 | Blount et al. 2012 |
| ZDB83 | 34,000 | C3+ | Blount et al. 2012 |
| ZDB87 | 34,000 | C2 | Blount et al. 2012 |
| ZDB96 | 36,000 | C3+H | Blount et al. 2012 |
| ZDB99 | 36,000 | C2 | Blount et al. 2012 |
| ZDB107 | 38,000 | C3+H | Blount et al. 2012 |
| ZDB111 | 38,000 | C2 | Blount et al. 2012 |
| REL10979 | 40,000 | C3+H | Blount et al. 2012 |
| REL10988 | 40,000 | C2 | Blount et al. 2012 |
| REL11364 | 50,000 | C3+H | Tenaillon et al. 2016 |
| REL11365 | 50,000 | C3+H | Tenaillon et al. 2016 |
