# Supplementary material for: Innovation in an E. coli evolution experiment is contingent on maintaining adaptive potential until competition subsides
Source: PLoS Genet. 2018 Apr 12;14(4):e1007348. doi: 10.1371/journal.pgen.1007348 (PMC5918244; doi:10.1371/journal.pgen.1007348)
Supplement: S2 Table — (DOCX) [file pgen.1007348.s007.docx]

| **Strain** | **Generation** | **Changed Allele** | **Difference** |
| --- | --- | --- | --- |
| ZDB409(+) | 5,000 | *ykgK*/*ykgL* | Evolved allele from ZDB478 added |
| ZDB409(+) | 5,000 | *eaeH* | Evolved allele removed |
| ZDB409(Ø)* | 5,000 | *eaeH* | Evolved allele removed |
| ZDB429(Ø)* | 10,000 | *ykgK*/*ykgL* | Evolved allele from ZDB478 added |
| ZDB446(+) | 15,000 | *ykgK*/*ykgL* | Evolved allele from ZDB478 added |
