# Supplementary material for: Innovation in an E. coli evolution experiment is contingent on maintaining adaptive potential until competition subsides
Source: PLoS Genet. 2018 Apr 12;14(4):e1007348. doi: 10.1371/journal.pgen.1007348 (PMC5918244; doi:10.1371/journal.pgen.1007348)
Supplement: S3 Table — Symbol legend: *for Ara+ revertant, (+) for Prnk-citT cassette, (Ø) for empty control cassette. (DOCX) [file pgen.1007348.s008.docx]

| **Strain** | **Generation** | **Description** |
| --- | --- | --- |
| DL82 | 0 | REL606(+) |
| DL86 | 0 | REL607(Ø)* |
| DL426 | 2,000 | REL1166A* |
| EQ966 | 2,000 | REL1166A(+) |
| DL631 | 2,000 | REL1166A(Ø)* |
| DL403 | 5,000 | ZDB409* |
| DL479 | 5,000 | ZDB409(+) |
| DL945 | 5,000 | ZDB409(Ø)* |
| DL418 | 10,000 | ZDB425* |
| DL439 | 10,000 | ZDB425(+) |
| DL959 | 10,000 | ZDB425(Ø)* |
| DL424 | 10,000 | ZDB429* |
| EQ972 | 10,000 | ZDB429(+) |
| DL480 | 10,000 | ZDB429(Ø)* |
| DL406 | 15,000 | ZDB446* |
| DL475 | 15,000 | ZDB446(+) |
| DL633 | 15,000 | ZDB446(Ø)* |
| DL415 | 20,000 | ZDB458* |
| DL13 | 20,000 | ZDB458(+) |
| DL376 | 20,000 | ZDB458(Ø)* |
| DL368 | 20,000 | ZDB464* |
| DL11 | 20,000 | ZDB464(+) |
| DL379 | 20,000 | ZDB464(Ø)* |
| DL366 | 20,000 | ZDB467* |
| DL12 | 20,000 | ZDB467(+) |
| DL377 | 20,000 | ZDB467(Ø)* |
| DL76 | 25,000 | ZDB478* |
| DL84 | 25,000 | ZDB478(+) |
| DL93 | 25,000 | ZDB478(Ø)* |
| DL363 | 25,000 | ZDB483* |
| DL15 | 25,000 | ZDB483(+) |
| DL374 | 25,000 | ZDB483(Ø)* |
| DL129 | 25,000 | ZDB486* |
| DL433 | 25,000 | ZDB486(+) |
| DL612 | 25,000 | ZDB486(Ø)* |
| DL137 | 27,000 | ZDB309* |
| DL158 | 27,000 | ZDB309(+) |
| DL159 | 27,000 | ZDB309(Ø)* |
| DL412 | 27,000 | ZDB310* |
| DL463 | 27,000 | ZDB310(+) |
| DL533 | 27,000 | ZDB310(Ø)* |
| DL409 | 27,000 | ZDB317* |
| DL471 | 27,000 | ZDB317(+) |
| DL957 | 27,000 | ZDB317(Ø)* |
| DL421 | 28,000 | ZDB334* |
| DL467 | 28,000 | ZDB334(+) |
| DL485 | 28,000 | ZDB334(Ø)* |
| DL359 | 28,000 | ZDB339* |
| EQ1104 | 28,000 | ZDB339(+) |
| DL370 | 28,000 | ZDB339(Ø)* |
| DL161 | 29,000 | ZDB13* |
| DL617 | 29,000 | ZDB13(+) |
| DL175 | 29,000 | ZDB13(Ø)* |
| DL361 | 29,000 | ZDB14* |
| EQ1068 | 29,000 | ZDB14(+) |
| DL372 | 29,000 | ZDB14(Ø)* |
| DL163 | 30,000 | ZDB18* |
| EQ1111 | 30,000 | ZDB18(+) |
| DL176 | 30,000 | ZDB18(Ø)* |
| DL164 | 30,500 | ZDB19* |
| EQ1113 | 30,500 | ZDB19(+) |
| DL180 | 30,500 | ZDB19(Ø)* |
| DL266 | 31,000 | ZDB23* |
| DL310 | 31,000 | ZDB23(+) |
| DL308 | 31,000 | ZDB23(Ø)* |
| DL167 | 31,500 | ZDB25* |
| DL201 | 31,500 | ZDB25(+) |
| DL185 | 31,500 | ZDB25(Ø)* |
| DL261 | 31,500 | ZDB27* |
| DL314 | 31,500 | ZDB27(+) |
| DL312 | 31,500 | ZDB27(Ø)* |
